# Cost-effectiveness Analysis of Japanese Encephalitis Vaccination for Children <15 Years of Age, Bangladesh

**DOI:** 10.3201/eid3012.231657

**Published:** 2024-12

**Authors:** An Nguyen, Rebeca Sultana, Elisabeth Vodicka, Zareen Tasnim, Kamran Mehedi, Md. Monjurul Islam, S.M. Abdullah Al Murad, Md. Redowan Ullah, Sharmin Sultana, Tahmina Shirin, Clint Pecenka

**Affiliations:** Program for Appropriate Technology in Health (PATH), Ho Chi Minh City, Vietnam (A. Nguyen); icddr,b, Dhaka, Bangladesh (R. Sultana, Z. Tasnim, M.R. Ullah); PATH, Seattle, Washington, USA (E. Vodicka, C. Pecenka); PATH, Dhaka (K. Mehedi); Maternal, Newborn, Child and Adolescent Health, Dhaka (M.M. Islam, S.M.A. Al Murad); Institute of Epidemiology Diseases Control and Research, Dhaka (S. Sultana, T. Shirin)

**Keywords:** Japanese encephalitis virus, vector-borne infections, meningitis/encephalitis, cost-effectiveness analysis, cost analysis, Japanese encephalitis vaccines, arboviruses, zoonoses, viruses, Bangladesh

## Abstract

Japanese encephalitis (JE) is preventable using the affordable, effective, and safe live attenuated SA 14-14-2 JE vaccine (CD-JEV). We used a Markov model to evaluate the cost-effectiveness of 1 dose of CD-JEV compared with no vaccination in 3 vaccination strategies in Bangladesh: subnational campaign and routine immunization, subnational campaign and national routine immunization, and national routine immunization alone. For input parameters, we gathered information from a cost-of-illness study, medical literature, government documents, and expert opinions. The base-case analysis estimated that a subnational campaign for children <15 years of age and routine immunization over 20 birth cohorts in Rajshahi, Rangpur, and Chattogram yielded (in 2021 US dollars) a cost of $82.2 million, $981/disability-adjusted life years averted, $9,964/case averted, and $49,819/death averted (societal perspective). We projected CD-JEV vaccination would be cost-effective across cost perspectives and vaccination strategies in Bangladesh, yielding an incremental cost-effectiveness ratio of approximately one third of per capita national gross domestic product.

Japanese encephalitis (JE) is a leading cause of viral encephalitis, particularly in endemic regions of the world; Asia bears a disproportionately high burden (*1*,*2*). JE virus is a mosquitoborne flavivirus that leads to acute encephalitis syndrome in an estimated 67,900 new clinical cases annually, mostly among children and young adults (*1*). JE virus kills ≈30% of those infected (*1*,*3*). Among survivors, ≈70% have long-term sequelae with possible motor, cognitive, and language impairments, as well as convulsions, behavioral problems, and mental disorders (*1*,*3*). No treatment for JE exists, but the disease can be effectively prevented with vaccines.

Since the first JE vaccine was developed in the 1930s, ≈15 additional vaccines with different technology platforms, presentations, and formulations have been added. Among them, the single-dose live attenuated SA14-14-2 JE vaccine (CD-JEV) has been the most widely used in JE-endemic countries; CD-JEV is affordable, effective, and safe (*4*,*5*). Over the past 3 decades, ≈350,000 children have been vaccinated with CD-JEV in 14 clinical safety trials, and 400 million doses have been administered (*6*). The seroprotection rates of CD-JEV in children vary from 80.2% to 99.1% in clinical trials (*7**–**9*). Vaccine effectiveness in endemic settings ranges from 80% to 99.3% at 6–15 months to 71% at 6 years after vaccination (*10**–**14*). 

In Bangladesh, the first JE outbreak was in 1977, but systematic assessment of disease occurrence was not conducted until hospital-based surveillance programs were implemented in 2003–2005 and 2007–2016 (*15**–**17*). Bangladesh has an annual incidence of 2.5 JE cases/100,000 persons <15 years of age, lower than in China (12.7), Korea (12.6), and Cambodia (10.6) (*1*). However, a recent cost-of-illness study in Bangladesh found that, despite the low incidence, JE remains a devastating disease because of its substantial economic burden (US$929/acute episode) and detrimental toll on the physical and psychosocial health of patients and their families (*18*). 

Over the past several decades, Bangladesh has made substantial progress in controlling vaccine-preventable diseases with high routine immunization coverage and introduction of new vaccines such as *Hemophilus influenzae* type B, rubella, pneumococcal conjugate vaccine, and inactivated polio vaccine (*19*). JE vaccine, however, has yet to be introduced into Bangladesh. In a multicriteria decision analysis by the World Health Organization (WHO), JE vaccine was ranked among the most highly recommended for introduction into Bangladesh (*20*), and several efforts have been made, including through advocacy workshops with decision-makers, to prioritize JE vaccine introduction in Bangladesh (*21**–**25*). A mass vaccination campaign including JE vaccine in the routine immunization program could have a profound effect on health outcomes. However, JE vaccine introduction may also create budget challenges for the government. Cost-effectiveness of and cost savings from JE vaccination has been demonstrated in many countries (*26**–**31*), but no such data exists for Bangladesh. We conducted this analysis to assess the effect and cost-effectiveness of CD-JEV vaccination compared with no vaccination among children in Bangladesh. We evaluated various vaccination strategies to inform the strategic decision-making of the government and international partners as they consider JE vaccine introduction and budgetary resources in the country. We obtained informed consent from the participants of this study. We conducted the study according to the guidelines of the institutional review board of icddr,b (Dhaka, Bangladesh), which approved the study protocol. 

## Materials and Methods

### Modeled Vaccination Strategies and Target Population

We met with policy makers, Expanded Program on Immunization (EPI) officers, international partners, and JE experts to identify vaccination strategies of interest. From this meeting, we identified 3 vaccination strategies to model. Strategy 1 (S1) is a subnational 1-time immunization campaign for children <15 years of age and subnational routine immunization for 9-month-old children. The subnational approach focused on 3 divisions with a high number of JE cases: Rangpur, Rajshahi, and Chattogram. Strategy 2 (S2) is a subnational 1-time immunization campaign and national routine immunization. Strategy 3 (S3) is national routine immunization only.

To have adequate time to observe immunization effectiveness on the population and explore changes in costs of the vaccination program, for each analysis strategy, we examined 10- and 20-year time horizons corresponding to consecutive 10 and 20 annual birth cohorts receiving vaccination. We adopted both governmental and societal cost perspectives. The societal cost perspective includes costs to the government, patients, and households to provide a complete picture of cost-effectiveness for healthcare payers in Bangladesh. The base case was the S1 strategy from a societal perspective for a 20-year time horizon. 

The target population for a JE vaccination campaign is children <15 years of age. On the basis of expert opinion, we assumed an average 5 years of age for children vaccinated during a JE campaign and a vaccination coverage rate of 88.6% on the basis of measles and rubella vaccine coverage rates in the EPI Coverage Evaluation Survey (*32*). The population for routine immunization is 9-month-old infants across 10 or 20 cohorts with an average annual birth rate of 1.81% as reported by the Bangladesh Bureau of Statistics. We sourced population sizes by divisions and age groups from Bangladesh 2022 census data. We used Bangladesh Bureau of Statistics life tables for background mortality rates in each age group to calculate non-JE deaths across participant life-cycles (*33*,*34*). 

For the campaign strategy, we summed outputs from the population of children 0–14 years of age. For the routine immunization strategy, we aggregated outputs of vaccinating 10 or 20 birth cohorts on the basis of current birth rates. We divided incremental costs for the vaccination compared with no-vaccination groups by incremental disability-adjusted life years (DALYs) to obtain the incremental cost-effectiveness ratio (ICER). We compared ICERs from different scenarios and presented findings as percentages of Bangladesh gross domestic product (GDP) per capita to evaluate the cost-effectiveness of CD-JEV in different vaccination strategies. 

### Model Framework

**Figure 1:**
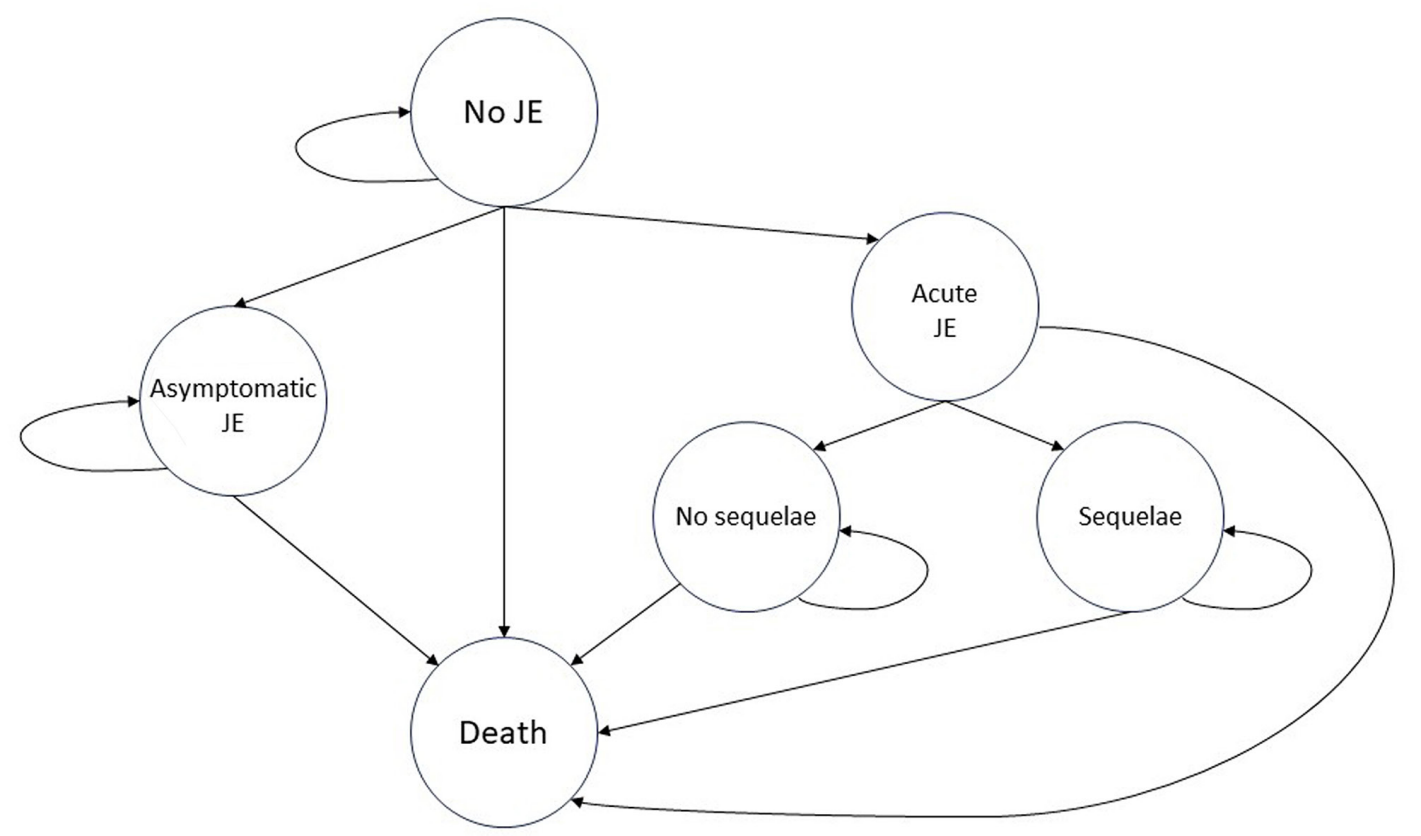
Markov model simulating costs and outcomes in cost-effectiveness analysis of JE vaccination for children <15 years of age, Bangladesh. All persons enter the model with no JE. Acute JE implies symptomatic JE and is a tunnel state, meaning any person in that state stays there for exactly 1 cycle. Those who had acute JE do have a higher mortality rate but must accrue the costs and disability-adjusted life years (DALYs) of the acute event before transitioning to death. Asymptomatic JE is not associated with higher mortality, costs, or DALYs; rather it eliminates any transition to acute JE. Costs and DALYs for acute and postacute JE are distributed by sequelae presence and severity. Vaccination changes the probability of transitioning from no JE to acute or asymptomatic JE. No other probabilities are changed by presence or absence of vaccination. Each state is associated with an annual cost and disability weight where applicable. This figure was remade from the Markov model in the study conducted in Philippines by the same research group from PATH (*31*). JE, Japanese encephalitis virus.

For the analysis, we used a previously developed cost-effectiveness model and input parameters gathered from global, regional, and Bangladesh national literature; published and unpublished government documents; and expert opinions from in-country public health leaders, immunization programs officers, and JE experts. We used the Microsoft Excel-based model (https://www.microsoft.com) that PATH developed and used in JE cost-effectiveness analyses for Indonesia and the Philippines (*26*,*30*). PATH developed a Markov model simulating costs and outcomes for children from time of vaccination or no vaccination until death or until reaching the time horizon of 100 years of age. Each child entered the model at a state of no JE and could remain in that state until death or transition to JE states, which included asymptomatic or acute JE (Figure 1). The probability of transition to acute JE was incidence in the no-vaccination group (comparator) and incidence multiplied by vaccine ineffectiveness rate in the vaccinated group (Table 1). Asymptomatic JE case-patients remained in this state, without any JE-associated costs or DALYs, until death. We assumed patients to have lifelong immunity because they did not transition to acute JE and could not return to the no-JE state. Acute JE case-patients accrued costs of care and quality of life decrements associated with an acute JE episode, then progressed to a postacute JE state with no, mild, moderate, or severe sequelae (on the basis of JE-associated chronic condition rates) or to death (on the basis of mortality rates) (Table 1). Case-patients in the postacute JE state remain in that state, with or without sequelae, until death. We modeled 1-year cycles as the time of transition between health states and distributed costs for sequelae care and DALYs by sequelae presence and severity (Table 1). 

**Table 1:** Summary of model parameters for Japanese encephalitis vaccination and epidemiology in Bangladesh, 2023*

Parameter	Base case estimate	Range	Source
Average age of vaccinated child in campaign, y	5	4–6	In-country expert opinion
Acute/symptomatic JE incidence, per 100,000 population	2.5	1.75–5.89	(*1*,*36*)
Asymptomatic-to-acute JE, ratio	300:1	25–1000:1	(*39*)
Case fatality/acute JE, %	20	10–30	(Institute of Epidemiology, unpub. data, email, 2023 Oct 03)
Duration of acute JE event	2.8 wks	2.2–3.3 wks	(*46*)
Sequelae incidence, %	58	46–70	(Institute of Epidemiology, unpub. data, email, 2023 Oct 03)
Sequelae severity, %			
Mild	16	13–19	(*18*)
Moderate	37	29–44	(*18*)
Severe	47	38–57	(*18*)
Probability of treatment by sequelae severity, %			
Mild	84	67–100	(*18*)
Moderate	89	72–100	(*18*)
Severe	96	77–100	(*18*)
Vaccine efficacy, %	86.3	69–98	(*9*,*12*)
Vaccine coverage, %	88.6	70.9–100	(*32*)
Discount rate for costs and health outcomes	3	NA	Assumption
Disability weight for acute JE, per event	0.133	0.088–0.190	Infectious disease acute episode–severe (*38*)
Disability weights for long-term sequelae, annual			
Mild	0.031	0.018–0.050	Motor/cognitive impairments–mild (*38*)
Moderate	0.203	0.134–0.290	Motor/cognitive impairments–moderate (*38*)
Severe	0.542	0.374–0.702	Motor/cognitive impairments–severe (*38*)
*JE, Japanese encephalitis; NA, not applicable.			

Among those progressing to JE, we assumed vaccination affected the probability of transition from the no-JE state to acute or asymptomatic JE but not duration or severity of JE and sequelae. We assumed no serious adverse event associated with JE vaccination but incorporated per-person costs associated with minor adverse events (Table 2). Main outputs included the number of JE cases averted, deaths averted, DALYs averted, costs of the vaccination program, total healthcare costs, and ICERs, calculated by dividing incremental costs by incremental DALY averted. We discounted costs and health outcomes by 3%/year using the common discount rate for lower-middle income countries and adjusted all monetary units amounts to 2021 US dollars (US $) (*35*). 

**Table 2:** Summary of model parameters for costs of Japanese encephalitis vaccine delivery and Japanese encephalitis treatment, Bangladesh, 2023*

Parameter	Base case estimate	Range	Source
Japanese encephalitis treatment costs, US $			
Japanese encephalitis-related hospitalization costs per event			
Direct medical cost to health system	106	77–135	(*18*)
Medical and non-medical cost to household	487	415–559	(*18*)
Indirect cost to household	351	254–448	(*18*)
Annual sequelae costs to health system			
Mild	6	0–14	(*18*)
Moderate	84	0–201	(*18*)
Severe	83	15–150	(*18*)
Annual sequelae costs to household			
Mild	213	71–355	(*18*)
Moderate	705	458–952	(*18*)
Severe	846	552–1,140	(*18*)
Vaccine-related costs			
Vaccine, adverse events, and supplies cost to government, per dose, US $	0.50	0.40–0.59	(*37*; S. Najibullah, unpub. data, internal report, 2023 Sep 16)
Annual increase in vaccine cost, %	5	4–6	Assumption
Vaccine delivery cost per dose for routine immunization, US $	1.71	1.37–2.05	(S. Najibullah, unpub. data, internal report, 2023 Sep 16)
Vaccine delivery cost per dose for campaign-based delivery, US $	1.10	0.88–1.32	(*41*)
Wastage, %	10	8–12	In-country expert opinion
Buffer, %	10	8–12	In-country expert opinion

*Costs are given in 2001 US dollars (US $).

### Sensitivity Analyses

To consider the uncertainty of model parameters, we conducted 1-way and probabilistic sensitivity analyses. In the 1-way sensitivity analysis, we varied each model parameter across a range of 95% CIs, or ±20% mean value, to observe how variation of each model input (e.g., sequelae incidence or vaccine efficacy) changed the ICER. To allow for exploring a wide range of JE incidence, we used 1.75–5.89 cases/100,000 persons <15 years of age on the basis of modeling estimates across divisions in Bangladesh (*36*). Using those variables, we defined key drivers of model outcomes as the parameters that had the largest effect on ICER. 

In the probabilistic sensitivity analysis, we explored the effect on model outcomes when jointly varying all model parameters over 10,000 Monte Carlo simulations. In each simulation, we randomly selected a value in every parameter’s distribution and used randomly selected values of all parameters to calculate model outputs. After conducting 10,000 simulations, we obtained the distribution of the mean and 95% CIs of model output values. We used a normal distribution for population size, age, duration of the acute JE event, and asymptomatic JE; γ distribution for costs; and β distribution for disability weights and percentages. We plotted results on a cost-effectiveness acceptability curve and evaluated them against willingness-to-pay thresholds to evaluate the likelihood that projected ICERs would be considered cost-effective in Bangladesh where no formal cost-effectiveness threshold has been established. 

### Model Outcomes and Costs

#### Health Outcomes 

The health outcomes we calculated were JE cases, deaths, expected life-years, and DALYs associated with acute JE episodes and different levels of sequelae. We calculated the number of JE cases based on JE incidence in children <15 years of age (2.5 cases/100,000 children) in low-incidence JE-endemic areas without a vaccination program, including Bangladesh (*1*). The vaccination group had fewer cases than the no-vaccination group because of the protective effect from CD-JEV calculated in the base case using a vaccine efficacy of 86.3% sourced from a clinical trial in Bangladesh (*9*). For sensitivity analysis, we used a 69%–98% efficacy range, the 95% CI for the pooled efficacy estimate from a systematic review of clinical trials (*12*). The mortality rate among symptomatic acute JE cases was 20% (S. Sultana, unpub. data, email, 2023 Oct 03). Among survivors, 58% had sequelae, among which 16% were mild, 37% moderate, and 47% severe, according to an unpublished estimation of 10 years of JE surveillance from the Institute of Epidemiology Diseases Control and Research (IEDCR) and a JE cost-of-illness study in Bangladesh (*18*,*37*). For DALYs associated with acute JE, we used disability weights for severe infectious disease acute episodes and for sequelae, and motor and cognitive impairments at mild, moderate, and severe levels from the literature (*38*). Those disability weights were used by WHO for the Global Burden of Disease study 2016, 2019, and 2021 editions and are the DALY weight descriptions that best match the symptoms of JE. For asymptomatic JE cases, which accrued no cost or disability weight, we used 300:1 for the rate of asymptomatic to symptomatic JE cases, as reported in the literature (*39*). 

#### Costs of Vaccination and Healthcare

We sourced costs of acute JE episodes and sequelae care from a JE cost-of-illness study that analyzed 10-year data from Bangladesh (*18*). We explored costs from governmental and societal perspectives. The governmental perspective considers direct medical costs (including for medicines, diagnostic tests, procedures, services, and facility fees) paid by the healthcare system to provide care for acute JE patients and those with sequelae. The societal perspective considers direct medical costs paid by the healthcare system and costs for the households including out-of-pocket medical costs, nonmedical costs (e.g., transportation, meals, and accommodations), and indirect costs for patients and caregivers, including income lost while caring for ill friends or family members (*18*). 

Expenses for the vaccination program were cost of the vaccine, including vaccine delivery during campaigns and routine immunization, supplies, and adverse events (Table 2). We used an assumed cost/CD-JEV dose in Bangladesh of US $0.44 on the basis of a reported 2023 price for Gavi-eligible countries (*37*). Supplies included unit costs of US $0.042/syringe and US $0.0056/syringe safety box (Najibullah S, unpub. data, internal report, 2023 Sep 16). According to WHO information, adverse events for CD-JEV vaccine include injection site reaction, fever, vomiting, abnormal crying, drowsiness, appetite loss, and irritability (*40*). There was no report on hypersensitivity reactions. We included an EPI-estimated adverse event cost of US $0.0081/dose for JE vaccine in the vaccination cost/dose (Government of Bangladesh, unpub. data, internal report, 2024 Aug 12). We estimated CD-JEV delivery costs in Bangladesh as US $1.71/dose for routine immunization (*40*) and US $1.10/dose during a vaccination campaign (*41*). We used rates of 10% for wastage and buffer and 5% for annual increases in vaccine cost based on expert opinion from the meeting with experts (policy makers, EPI officers, international partners, and JE experts). 

## Results 

For base analysis S1 (subnational campaign and subnational routine immunization in Rangpur, Rajshahi, and Chattogram divisions), we estimated that the discounted vaccine program costs borne by the government would be US $82.2 million for 20 years but would save households and the government of Bangladesh US $75.1 million in healthcare costs. We calculated the S1 vaccination strategy would prevent 7,544 JE cases and 1,509 deaths (Table 3) and yield ICERs of US $94/DALY averted, $958/case averted, and $4,790/death averted from the societal perspective and $981/DALY averted, $9,964/case averted, and $49,819/death averted from the governmental perspective (Table 4). The costs/DALY averted correspond to 3.5%–36.6% of Bangladesh 2021 GDP per capita. Estimating results from S1 with a shorter time horizon, 1-time campaign, and 10 years of routine immunization led to lower costs but fewer health outcomes averted because fewer children (≈12 million) would be vaccinated (Tables 3, 4). The cost/DALY averted varied within 0.8%–33.3% of Bangladesh GDP per capita, depending on societal or governmental perspective. 

**Table 3:** Discounted incremental outcomes and program costs of different Japanese encephalitis vaccination strategies as compared with no vaccination from the governmental payer and societal perspectives in Bangladesh

Discounted incremental outcomes	Routine immunization birth cohorts	Strategy 1, subnational campaign + subnational routine*	Strategy 2, subnational campaign + national routine†	Strategy 3, national routine‡
Cases averted	10	5,733	8,962	5,663
	20	7,544	13,176	9,876
DALYs averted	10	58,130	91,104	57,829
	20	76,624	134,134	100,859
Deaths averted	10	1,147	1,792	1,133
	20	1,509	2,635	1,975
Total vaccinated	10	30,337,406	45,866,646	27,234,783
	20	42,042,950	73,101,429	54,469,566
Discounted vaccine program costs, $§	10	57.1M	95.5M	67.3M
	20	82.2M	154.0M	125.8M
*Subnational 1-time immunization campaign for children <15 years of age and subnational routine immunization for 9-month-old children. The subnational approach focuses on 3 divisions with a high number of JE cases: Rangpur, Rajshahi, and Chattogram. †Subnational 1-time immunization campaign and national routine immunization. ‡S3, national routine immunization only. §US dollars.				

**Table 4:** Discounted incremental costs of different Japanese encephalitis vaccination strategies as compared with no vaccination from the governmental payer and societal perspectives in Bangladesh*

Discounted incremental outcomes	Routine immunization birth cohorts	Strategy 1, subnational campaign + subnational routine†			Strategy 2, subnational campaign + national routine‡			Strategy 3, national routine§	
		Govt	Soc		Govt	Soc		Govt	Soc
Discounted healthcare costs averted through vaccination	10	5.3M	55.8M		8.5M	90.1M		5.7M	60.1M
	20	7.1M	75.1M		12.7M	134.8M		9.9M	104.8M
Deterministic ICER: cost per DALY averted	10	892	23		955	60		1,066	126
	20	981	94		1,053	143		1,149	208
Deterministic ICER: cost per case averted	10	9,046	230		9,710	609		10,888	1,283
	20	9,964	958		10,720	1,458		11,733	2,128
Deterministic ICER: cost per death averted	10	45,230	1,148		48,549	3,045		54,441	6,413
	20	49,819	4,790		53,601	7,290		58,667	10,640
Probabilistic ICER: cost per DALY averted, mean (95% CI)	10	991 (358–2,272)	173 (–632 to 1,719)		1,351 (453–3,469)	196 (–501 to 2,614)		1,374 (439–3,557)	445 (–644 to 2,430)
	20	1,198 (444 to 2,807)	307 (–538 to 1,971)		1,249 (352 to 2,684)	377 (–593 to 2,520)		1,394 (559 to 2,834)	560 (–475 to 2,879)
*Values are in US dollars. DALY, disability-adjusted life year; ICER, incremental cost-effectiveness ratio; govt, governmental costs; soc, societal costs. †Subnational 1-time immunization campaign for children <15 years of age and subnational routine immunization for 9-month-old children. The subnational approach focuses on 3 divisions with a high number of JE cases: Rangpur, Rajshahi, and Chattogram. ‡Subnational 1-time immunization campaign and national routine immunization. §National routine immunization only.									

For S2, with CD-JEV included as part of the routine immunization program implemented nationwide instead of only subnationally in 3 divisions with high endemicity, as was the plan in S1, vaccine program costs increased to US $154 million over 20 years. However, S2 would save society US $134.8 million in downstream healthcare and household costs from 13,176 JE cases and 2,635 deaths averted. S2 strategy had ICERs of US $143/DALY averted, $1,458/case averted, and $7,290/death averted from a societal perspective and $1,053/DALY averted, $10,720/case averted, and $53,601/death averted from a governmental perspective (Table 4). With a higher overall ICER than S1, the S2 vaccination strategy was less cost-effective.

S3 (standalone national routine immunization) resulted in fewer vaccinated children than in S1, the base case scenario, over a 10-year period but more children over a 20-year period (Table 3). The S3 strategy had higher program costs than S1, regardless of the time horizon. It also had the highest ICER among the 3 vaccination strategies, with US $208/DALY averted, $2,128/case averted, and $10,640/death averted from a societal perspective and $1,149/DALY averted, $11,733/case averted, and $58,667/death averted from a governmental perspective (Table 4). Thus, S3 was the least cost-effective among the 3 vaccination strategies (Appendix Table). 

**Figure 2:**
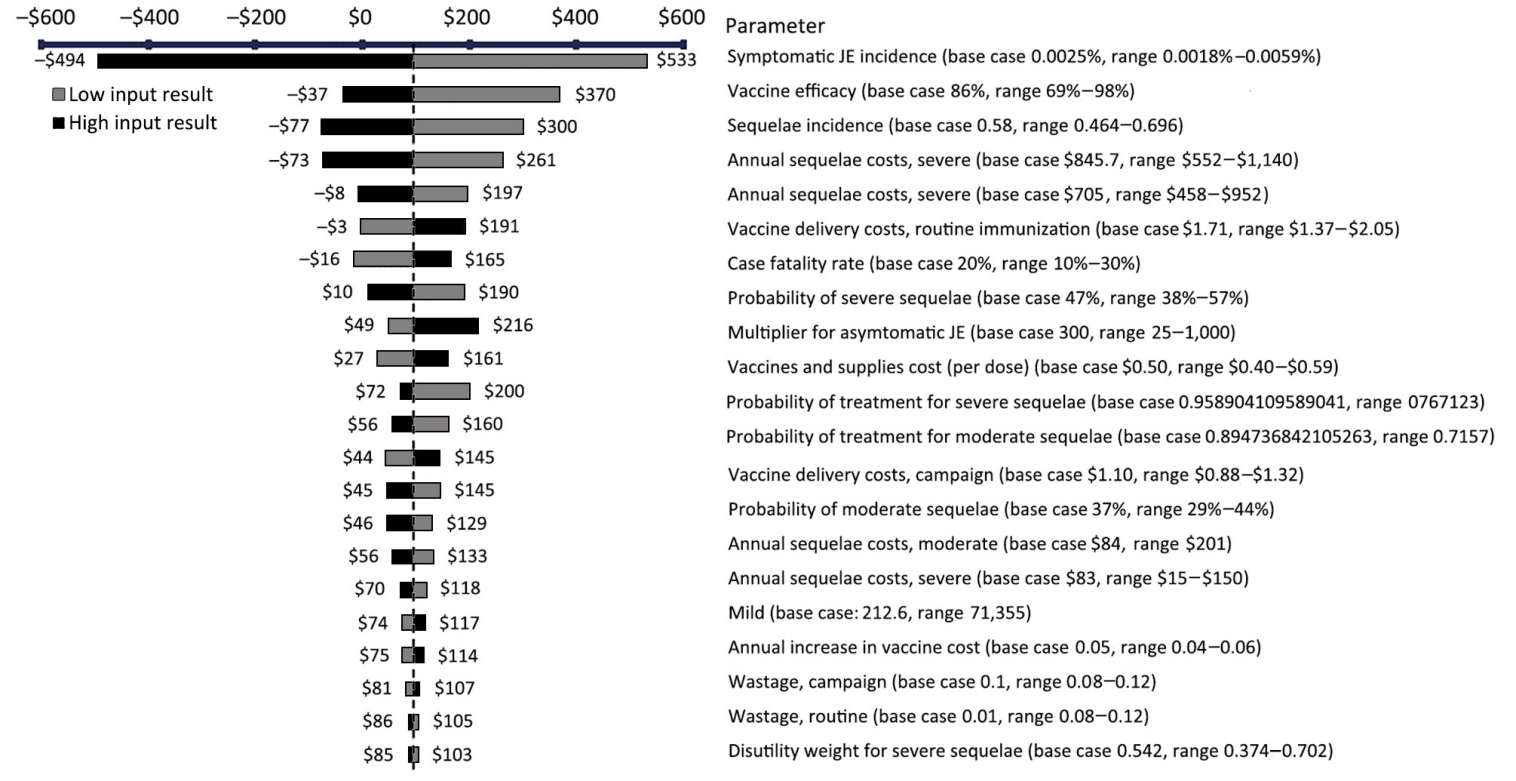
Societal perspective for 1-way sensitivity analysis of key cost drivers for cost per DALY averted with strategy 1 in cost-effectiveness analysis of JE vaccination for children <15 years of age, Bangladesh. Strategy 1 consisted of a subnational 1-time immunization campaign for children <15 years of age and subnational routine immunization for 9-month-old children over 20 birth cohorts. The subnational approach focuses on 3 divisions with a high number of JE cases: Rangpur, Rajshahi, and Chattogram. Values are US dollars. JE, Japanese encephalitis virus.

**Figure 3:**
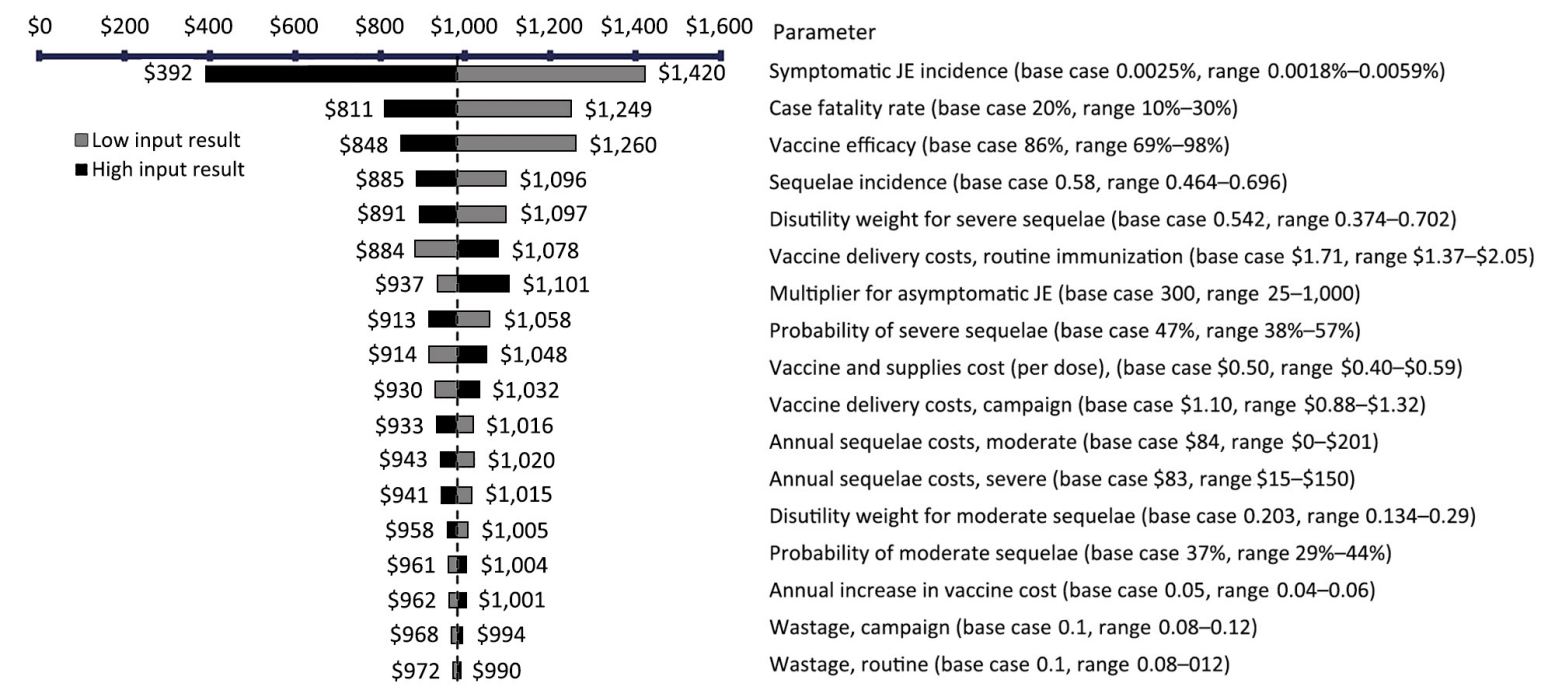
Governmental perspective for 1-way sensitivity analysis of key cost drivers for cost per DALY averted with strategy 1 in cost-effectiveness analysis of JE vaccination for children <15 years of age, Bangladesh. Strategy 1 consisted of a subnational 1-time immunization campaign for children <15 years of age and subnational routine immunization for 9-month-old children over 20 birth cohorts. The subnational approach focuses on 3 divisions with a high number of JE cases: Rangpur, Rajshahi, and Chattogram. Values are US dollars. JE, Japanese encephalitis virus.

One-way sensitivity analysis results (shown as tornado plots) identified the parameters most influencing cost/DALY averted (Figures 2, 3). The strongest cost drivers from the societal perspective were symptomatic JE incidence, vaccine efficacy, sequelae incidence, and sequelae costs. From the governmental perspective, the strongest cost drivers were case-fatality rate, symptomatic JE incidence, vaccine efficacy, and sequelae incidence. When these key cost drivers were adjusted higher or lower, ICERs varied from negative (cost saving) values to the highest value of US $1,420/DALY for S1, $1,521/DALY for S2, and $1,655/DALY for S3 from the governmental perspective; social perspectives produce lower ICERs. Maximum ICERs corresponded to 53% of the Bangladesh GDP per capita for S1, 57% for S2, and 62% for S3. 

**Figure 4:**
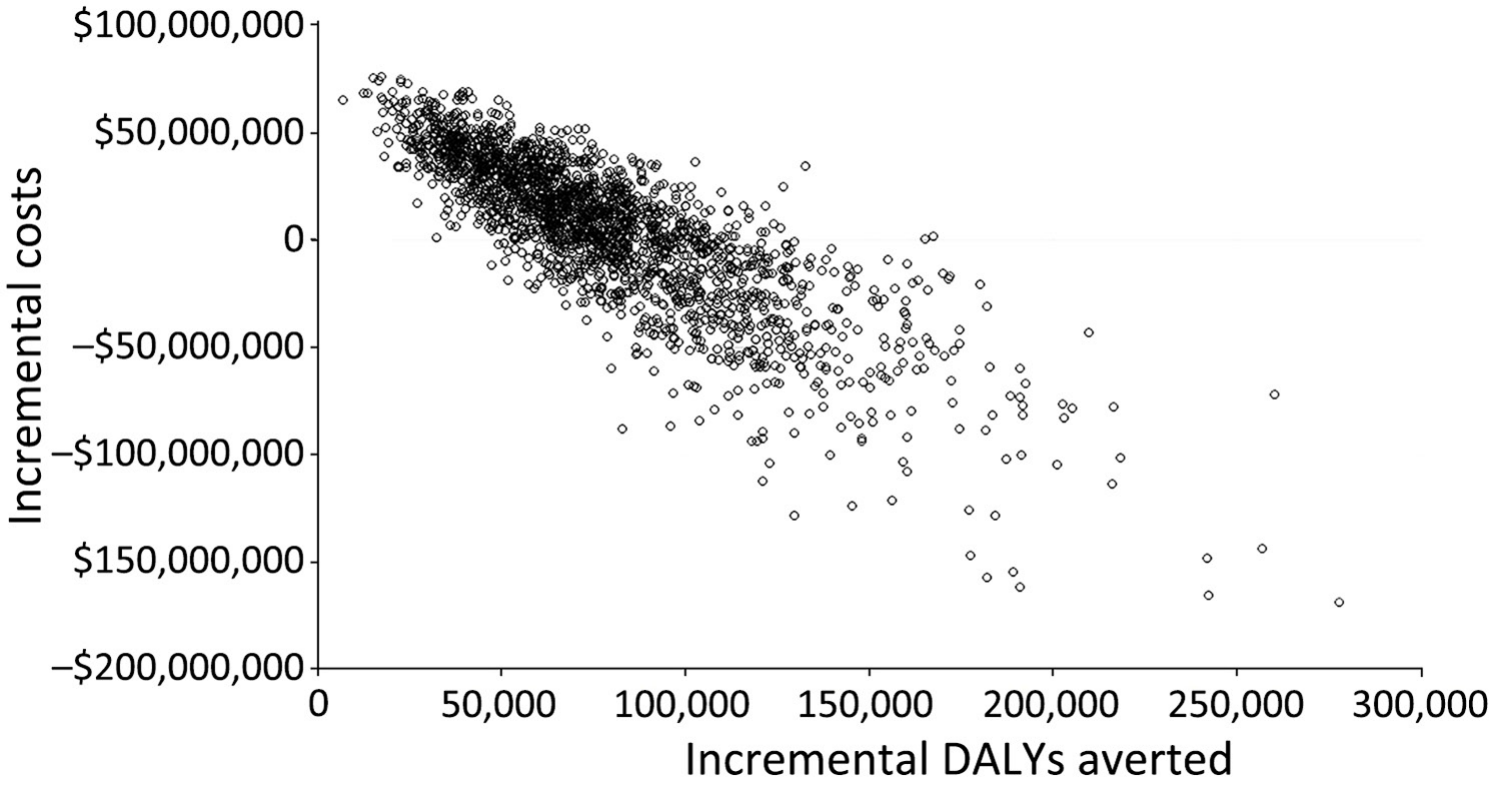
Societal perspective for probabilistic results of key cost drivers for cost per DALY averted with strategy 1 in cost-effectiveness analysis of JE vaccination for children <15 years of age, Bangladesh. Strategy 1 consisted of a subnational 1-time immunization campaign for children <15 years of age and subnational routine immunization for 9-month-old children over 20 birth cohorts. The subnational approach focuses on 3 divisions with a high number of JE cases: Rangpur, Rajshahi, and Chattogram. Values are US dollars. JE, Japanese encephalitis virus.

**Figure 5:**
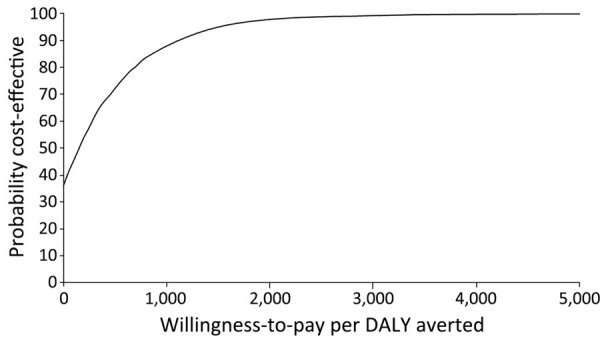
Cost-effectiveness of strategy 1 in cost-effectiveness analysis of JE vaccination for children <15 years of age, Bangladesh. Strategy 1 consisted of a subnational 1-time immunization campaign for children <15 years of age and subnational routine immunization for 9-month-old children over 20 birth cohorts. The subnational approach focuses on 3 divisions with a high number of JE cases: Rangpur, Rajshahi, and Chattogram. This strategy would be considered cost-effective in 99% of simulations at a willingness-to-pay threshold of US $2,400/DALY averted, which is <1 times gross domestic product per capita. DALY, disability-adjusted life year; JE, Japanese encephalitis virus.

When we varied all parameters in their distribution for the probabilistic sensitivity analysis, our results were robust. Probabilistic ICERs were close to deterministic ICERs (Table 4). We performed 10,000 simulations to calculate incremental costs and DALYs averted in which we randomly selected all model parameters from their distribution (Figure 4). In many simulations, the subnational campaign (S1) and national routine immunization (S2) were cost-saving from the societal perspective through 20 birth cohorts. When we compared ICER results with potential willingness-to-pay thresholds in Bangladesh, we projected that the S1 vaccination strategy of subnational campaign plus subnational routine immunization from the societal perspective over 20 birth cohorts would be considered cost-effective in 99% of simulations at a willingness-to-pay threshold of US $2,400/DALY averted, which is <1 times GDP per capita (Figure 5). 

## Discussion

Our findings show that introducing CD-JEV vaccination could avert JE cases and deaths and improve the quality of life for the at-risk population in Bangladesh. In addition, we show that vaccine program costs are offset by large cost savings from healthcare services and costs to families. CD-JEV appears to be cost-effective because ICERs were approximately one third GDP per capita, regardless of vaccination strategy or cost perspective. 

Our projection was robust in deterministic and probabilistic analyses in which we varied model parameters to account for uncertainties. We applied a relatively conservative estimate of vaccine efficacy (86.3%), one of the key drivers of cost/DALY averted. Estimates in the literature report a higher vaccine efficacy of 93% based on pooled odds ratios from 5 case–control studies evaluating the effectiveness of a live attenuated SA14-14-2 JE vaccine (*10*). We evaluated the potential impact of higher vaccine efficacy (up to 98%) through sensitivity analysis with ICER results ranging from cost saving to US $1,260/DALY averted. Varying input parameters across their uncertainty ranges still showed robust evidence of cost-effectiveness with the maximum ICERs corresponding to 52%–62% GDP per capita. Without an established cost-effectiveness threshold in Bangladesh, CD-JEV vaccination is projected to be 99% cost-effective at a willingness-to-pay level of <US $2,676 or 1 GDP per capita and is projected to be 100% cost-effective at 1–3 times GDP per capita willingness-to-pay. 

The large difference between governmental- and societal-perspective ICERs (US $892–$981 vs. $23–$94/DALY averted) in our analysis reflects that the cost of JE illness in Bangladesh is borne largely by patients and their families (*18*). Our findings on the cost-effectiveness of CD-JEV immunization is similar to other studies (*26*,*29**–**31*,*42*). However, the burden of disease borne by families, reflected by differences in governmental and societal ICERs, is more substantial in Bangladesh, where governmental:societal ICER ratio is >10:1, than in Indonesia, where the ratio is ≈2:1 (*27*), or the Philippines, where the ratio varies from 4:1 to 9:1 (*30*). 

A limitation of our study was lack of epidemiologic evidence for JE incidence in Bangladesh. We used the symptomatic JE incidence of 2.5 cases/100,000 persons <15 years of age sourced from a systematic review of studies from many countries, including Bangladesh (*3**1*). However, the data are from 2011 and might not reflect the most current JE status in the country. In the sensitivity analysis, we used a different source of incidence, taken from a modeling study in 2014 based on a range of incidence across divisions in Bangladesh (1.75–5.89/100,000 persons <15 years of age) (*36*). Our analysis might have underestimated the true number of JE cases, because numerous encephalitis patients never participated in the hospital-based surveillance program because of long distance (*43*), limited access to healthcare services in rural Bangladesh (*44*), or unwillingness to receive care from hospitals (*45*). Furthermore, we depended on expert guidance for our vaccine coverage rate in the absence of available evidence. Nevertheless, in a country like Bangladesh, where there is scant evidence available on incidence, outcomes, and costs associated with JE, our study can contribute to introducing and expanding the JE vaccine program. 

Our study provided evidence of CD-JEV cost-effectiveness for different vaccination strategies and supports value-based decision-making for JE vaccine introduction in Bangladesh. We project that subnational or national routine immunization, combined with a one-time subnational introductory campaign, would be more cost-effective than national routine immunization alone. That projection aligns with the WHO position that the most effective JE vaccine introduction strategy is a one-time campaign followed by adding JE vaccination to routine immunization practices (*4*). Both S1 and S2 vaccination strategies were cost-effective with maximum ICERs at the WTP level of ≈50% of GDP per capita. A 10-year vaccination program using the S2 strategy could, at a cost of an additional US $38 million, immunize >15 million more children than the S1 strategy.

Our study adds more evidence on the cost-effectiveness of CD-JEV in general and provides cost-effectiveness data on the various vaccination strategies. These findings have strong policy implications in Bangladesh, where policymakers are considering JE-vaccine introduction, and will help determine resource availability and introduction strategies to maximize public health impact from and cost-effectiveness of CD-JEV. 

Appendix Additional information from study of cost-effectiveness analysis of Japanese encephalitis vaccination for children <15 years of age in Bangladesh.
