# Supplementary material for: Cost-effectiveness Analysis of Japanese Encephalitis Vaccination for Children <15 Years of Age, Bangladesh
Source: Emerg Infect Dis. 2024 Dec;30(12):2593–603. doi: 10.3201/eid3012.231657 (PMC11616670; doi:10.3201/eid3012.231657)
Supplement: Appendix — Additional information from study of cost-effectiveness analysis of Japanese encephalitis vaccination for children <15 years of age in Bangladesh. [file 23-1657-Techapp-s1.pdf]

Article DOI: https://doi.org/10.3201/eid3012.231657

EID cannot ensure accessibility for supplementary materials supplied by authors. Readers who have difficulty accessing supplementary content should contact the authors for assistance.

Cost-Effectiveness Analysis of Japanese Encephalitis Vaccination for Children <15 Years of Age, Bangladesh

Appendix

Appendix Table. Comparisons of incremental costs and outcomes of 3 vaccination strategies over 20 years*

	Difference between S2 and S1 (S2–S1)		Difference between S3 and S1 (S3–S1)		Differences between S3 and S2 (S3–S2)	
Discounted incremental outcomes	Governmental	Societal	Governmental	Societal	Governmental	Societal
Cases averted, n	5,632	NA	2,332	NA	–3,300	NA
DALYs averted, n	57,510	NA	24,235	NA	–33,275	NA
Deaths averted, n	1,126	NA	466	NA	–660	NA
Total vaccinated, n	31,058,479	NA	12,426,616	NA	–18,631,863	NA
Discounted vaccine program costs, USD, millions	71.8	NA	43.6	NA	–28.2	NA
Discounted healthcare costs averted by vaccination strategy, USD millions	5.6	59.7	2.8	4.3	–2.8	–30
Deterministic ICER: cost/DALY averted, USD	72/DALY	49/DALY	168/DALY	114/DALY	96/DALY	65/DALY
Deterministic ICER: cost/case averted, USD	756/case	500/case	1,769/case	1,170/case	1,013/case	670/case
Deterministic ICER: cost/death averted, USD	3,782/death	2,500/death	8,848/death	5,850/death	5,066/death	3,350/death

*DALY, disability-adjusted life years; NA, not applicable; S1, subnational campaign and subnational routine immunization strategy; S2, subnational campaign and national routine immunization strategy; S3, national routine immunization strategy; USD, US dollars (2021)
